# Cost-effectiveness of sequential daily teriparatide/weekly alendronate compared with alendronate monotherapy for older osteoporotic women with prior vertebral fracture in Japan

**DOI:** 10.1007/s11657-021-00891-z

**Published:** 2021-04-17

**Authors:** Takahiro Mori, Carolyn J. Crandall, Tomoko Fujii, David A. Ganz

**Affiliations:** 1 Department of Health Services Research, Faculty of Medicine, University of Tsukuba, Tsukuba, Ibaraki Japan; 2 Health Services Research and Development Center, University of Tsukuba, Tsukuba, Ibaraki Japan; 3 Department of General Internal Medicine, Eastern Chiba Medical Center, Togane, Chiba Japan; 4 Division of General Internal Medicine and Health Services Research, Department of Medicine, David Geffen School of Medicine at University of California, Los Angeles, Los Angeles, CA USA; 5 Department of Medical Research and Management for Musculoskeletal Pain, 22nd Century Medical & Research Center, The University of Tokyo Hospital, Tokyo, Japan; 6 Geriatric Research, Education and Clinical Center and HSR&D Center for the Study of Healthcare Innovation, Implementation and Policy, Veterans Affairs Greater Los Angeles Healthcare System, Los Angeles, CA USA; 7 Division of Geriatrics, Department of Medicine, David Geffen School of Medicine at University of California, Los Angeles, Los Angeles, CA USA; 8 Health Unit, RAND Corporation, Santa Monica, CA USA

**Keywords:** Cost-effectiveness analysis, Osteoporosis, Fracture prevention, Teriparatide, Biosimilar

## Abstract

***Summary*:**

Using a Markov microsimulation model among hypothetical cohorts of community-dwelling older osteoporotic Japanese women with prior vertebral fracture over a lifetime horizon, we found that daily subcutaneous teriparatide for 2 years followed by weekly oral alendronate for 8 years was not cost-effective compared with alendronate monotherapy for 10 years.

**Purpose:**

Teriparatide has proven efficacy in reducing osteoporotic fractures, but with substantial cost. We examined the cost-effectiveness of sequential teriparatide/alendronate (i.e., daily subcutaneous teriparatide for 2 years followed by weekly oral alendronate for 8 years) compared with alendronate monotherapy for 10 years among community-dwelling older osteoporotic women with prior clinical or morphometric vertebral fracture in Japan.

**Methods:**

Using a previously validated and updated Markov microsimulation model, we obtained incremental cost-effectiveness ratios (Japanese yen [¥] (or US dollars [$]) per quality-adjusted life year [QALY]) from the perspective of a single payer responsible for both public healthcare and long-term care. We assumed a lifetime horizon with a willingness-to-pay of ¥5million (or $47,500) per QALY in the base case. We modeled the cost of biosimilar teriparatide, which has been available since November 2019 in Japan, assuming the efficacy was the same as that of the brand version.

**Results:**

In the base case, sequential teriparatide/alendronate was not cost-effective compared with alendronate monotherapy. In deterministic sensitivity analyses, sequential teriparatide/alendronate would become cost-effective with 85%, 50%, and 15% price discounts to teriparatide at ages 70, 75, and 80, respectively, compared to the current biosimilar cost. Otherwise, results were especially sensitive to changes that affected efficacy of teriparatide or alendronate. In probabilistic sensitivity analyses, the probabilities of sequential teriparatide/alendronate being cost-effective were 0%, 1%, and 37% at ages 70, 75, and 80, respectively.

**Conclusions:**

Among high-risk osteoporotic women in Japan, sequential teriparatide/alendronate was not cost-effective compared with alendronate monotherapy, even with the availability of biosimilar teriparatide.

**Supplementary Information:**

The online version contains supplementary material available at 10.1007/s11657-021-00891-z.

## Introduction

Osteoporosis leads to fragility fractures and constitutes a major medical and public health concern worldwide. Vertebral fracture is one of the most common fragility fractures and can be symptomatic (i.e., clinical vertebral fracture) or asymptomatic (i.e., morphometric vertebral fracture). Clinical vertebral fracture can lead to significant morbidity and loss of quality of life [1]. Compared with Caucasians, the Japanese population has been reported to have higher annual incidence rates of clinical vertebral fracture and lower rates of hip fracture [2, 3]. This makes a strategy to reduce the risk of vertebral fracture important in the Japanese population, especially in those at high risk for vertebral fracture, such as those with a prior history of vertebral fracture [4].

Teriparatide is a recombinant parathyroid hormone that stimulates bone formation and activates bone remodeling, and was the first anabolic agent to become available for the treatment of osteoporosis. The current Japanese guidelines for the prevention and treatment of osteoporosis conclude that there is high-quality evidence for teriparatide increasing bone mineral density and reducing the risk of vertebral fracture. The guidelines recommend that teriparatide generally should not be used as a first-line drug for the treatment of osteoporosis, but could be considered a treatment of choice for those at high risk for osteoporotic fracture [5].

Teriparatide’s expense is one of its main disadvantages. Indeed, brand versions of teriparatide (i.e., daily and weekly versions available in Japan) are the most expensive medications for the treatment of osteoporosis in Japan and consisted of 20% of the total costs (i.e., the sums of payments by third-party payers and by patients out-of-pocket) for medications for osteoporosis in Japan in 2017, followed by vitamin D supplementation (35%) and bisphosphonates (32%) [6]. Biosimilar daily teriparatide has been available in Japan since November 2019, and the initial price of the biosimilar version was set to be 60% of the equivalent brand product [7]. Biosimilar drugs are biological products that are highly similar to the approved biologic reference products and have no clinically meaningful differences from the reference products [8].

In our previous study in the US setting, we examined the potential health economic impact of generic or biosimilar (i.e., generic/biosimilar) daily teriparatide availability after the market exclusivity for brand teriparatide expired in August 2019 in the USA. We found that among high-risk older osteoporotic white women, even with generic/biosimilar teriparatide availability, teriparatide followed by alendronate (i.e., sequential teriparatide/alendronate) would not be cost-effective unless the cost of generic/biosimilar teriparatide was heavily discounted with respect to the current brand cost [9].

To the best of our knowledge, a cost-effectiveness analysis including teriparatide has not yet been conducted in Japan. Generating a specific cost-effectiveness analysis for Japan is important, because results of cost-effectiveness analyses performed for one country may not apply to another country with a different disease epidemiology, healthcare system costs, and willingness-to-pay threshold. With the recent availability of the less expensive biosimilar version of teriparatide in Japan, we therefore aimed to examine the cost-effectiveness of including teriparatide as part of osteoporosis treatment in the Japanese setting.

As a typical scenario for teriparatide’s use, we compared the cost-effectiveness of sequential teriparatide/alendronate, which in this study was defined as daily subcutaneous teriparatide for 2 years followed by weekly oral alendronate for 8 years, compared with weekly oral alendronate monotherapy for 10 years among women with prior clinical or morphometric vertebral fracture in Japan. Worldwide, teriparatide is approved for no more than a 2-year treatment period because of the potential risk of osteosarcoma observed in animal studies [10]. Bisphosphonates are typically prescribed after the completion of teriparatide to prevent decline in bone mineral density and provide continued fracture prevention [9]. Although the optimal duration of alendronate has not been determined, those who are at high risk for osteoporotic fractures may benefit from more than 5 years, and up to 10 years of therapy [5, 11–14].

## Materials and methods

### Overview

We updated a Markov microsimulation model, which was built based on our previous work and validated [9, 15, 16], to perform a cost-effectiveness analysis among hypothetical cohorts of community-dwelling osteoporotic women in Japan at various ages of therapy initiation (70, 75, and 80 years). We estimated quality-adjusted life years (QALYs) and total costs in 2020 Japanese yen (¥). For ease of interpretation, we converted these results to US dollars ($) at a rate of ¥105 to $1, which approximates the current exchange rate as of November 2020 [17]. We obtained incremental cost-effectiveness ratios (ICERs) over a lifetime horizon (until a participant reached age 105 years, or died). We evaluated cost-effectiveness from the perspective of a single payer responsible for both public healthcare and long-term care (including public healthcare costs covered by public healthcare insurance and public long-term care costs covered by long-term care insurance in Japan) in the base case and deterministic and probabilistic sensitivity analyses [18]. In addition, the public healthcare payer’s perspective (including public healthcare costs, but not including public long-term care costs) was adopted as a sub-analysis of the base case (Supplemental Table 1). We did not include the societal perspective [18].

We set a willingness-to-pay threshold of ¥5 million ($47,500) per QALY in the base case [15]. In deterministic and probabilistic sensitivity analyses, we also set a willingness-to-pay threshold of ¥10 million ($95,000) per QALY (as an upper bound of willingness-to-pay) in addition to the threshold of ¥5 million per QALY. The upper bound of willingness-to-pay was based on a cut-point that the Central Social Insurance Medical Council (i.e., Chuikyou in Japanese) proposed to establish the most expensive tier of selected new pharmaceuticals and medical devices when conducting health technology assessment in Japan [19]. We discounted all costs and health benefits at 2% per year for the base case [18].

An extensive systematic review was performed for all the parameters in the model, and inputs were derived from peer-reviewed literature (e.g., meta-analyses of randomized controlled trials, observational studies, and cost-effectiveness analyses), and websites (e.g., statistics reports from the Ministry of Health, Labour and Welfare, drug prices, and currency exchange rate) that were considered most relevant (e.g., Japanese population), high-quality, and up-to-date estimates. Our own assumptions were chosen only if no reliable published estimate was available (Table 1).

**Table 1:** Model parameters

	Value	Range for deterministic sensitivity analysis	Distribution and range for probabilistic sensitivity analysis	Reference
Teriparatide				
Relative risk of hip fracture	0.35	0.15–0.73a	Beta: 0.15–0.73^a^	[20]
Relative risk of clinical vertebral fracture	0.23	0.16–0.32^a^	Beta: 0.16–0.32^a^	[20]
Adherence rate (first year)	0.70	0.6–0.8^b, c^	Triangular: 0.6–0.8^b, c^	[21]
Persistence rate (first year)	0.68	± 15%^b^ for annual rate	Triangular: ± 15%^b^ for annual rate	[22]
Treatment duration (years)	2	N/A	N/A	[10]
Offset effect (years)	2	3^d^	N/A	[23]
Alendronate				
Relative risk of hip fracture	0.64	0.45–0.88^a^	Beta: 0.45–0.88^a^	[20]
Relative risk of clinical vertebral fracture	0.50	0.40–0.64^a^	Beta: 0.40–0.64^a^	[20]
Adherence rate (first year)	0.71	0.62–0.8^b, c^	Triangular: 0.62–0.8^b, c^	[21]
Persistence rate (first year)	0.55	± 15%^b^ for annual rate	Triangular: ± 15%^b^ for annual rate	[21]
Treatment duration (years)	8 or 10	N/A	N/A	[11–14]
Offset effect (years)	Same as above	N/A	N/A	[9, 15, 16]
Costs, ¥ (US dollars, $1=¥105)				
Annual medication costs and costs of prescription charges at pharmacy				
Teriparatide	¥333,400 ($3180)	decreased in 5% increments	N/A	[7]
Alendronate	¥8700 ($83)	N/A	N/A	[7]
Prescription charge for teriparatide	¥1100 ($10)	N/A	N/A	[24]
Prescription charge for alendronate	¥1700 ($16)	N/A	N/A	[24]
Costs for physician visits, blood test and DXA scan				
First physician visit, teriparatide	¥18,300 ($174)	N/A	N/A	[24]
Subsequent physician visit, teriparatide	¥9400 ($90)	N/A	N/A	[24]
First physician visit, alendronate	¥3600 ($34)	N/A	N/A	[24]
Subsequent physician visit, alendronate	¥1900 ($18)	N/A	N/A	[24]
Blood test	¥2900 ($28)	N/A	N/A	[24]
DXA scan	¥4500 ($43)	N/A	N/A	[24]
Medical costs				
Hip fracture	¥1,726,000 ($16,440)	± 50%^b^	Triangular: ± 50%^b^	[25]
First clinical vertebral fracture	¥420,000 ($4000)	± 50%^b^	Triangular: ± 50%^b^	[25]
Subsequent clinical vertebral fracture	¥842,000 ($8020)			[25]
Annual long-term care costs				
For the “post-hip fracture” state	¥876,000 ($8340)	± 50%^b^	Triangular: ± 50%^b^	[26]
For the “post-vertebral fracture” state	¥213,000 ($2030)	± 50%^b^		[25, 26]
Utilities				
Age 65–69	0.862	N/A	Triangular: ± 15%^b^	[27]
Age 70–74	0.810	N/A		
Age 75–79	0.771	N/A		
Age 80–84	0.769	N/A		
Age 85+	0.684	N/A		
Disutilities (multiplier)				
Hip fracture, first year	0.776	N/A	Beta: 0.720–0.844^a^	[28, 29]
Hip fracture, beyond first year	0.855	N/A	Beta: 0.800–0.909^a^	[28, 29]
Clinical vertebral fracture, first year	0.724	N/A	Beta: 0.667–0.779^a^	[28, 29]
Clinical vertebral fracture, beyond first year	0.868	N/A	Beta: 0.827–0.922^a^	[28, 29]
Annual hip fracture incidence rates per 100,000 persons (without intervention)				
Age 70–74	158.1	± 50%^b^	Triangular: ± 10%^b^	[30]
Age 75–79	362.2			
Age 80–84	851.1			
Age 85–89	1580.2			
Age 90–94	2466.0			
Age 95–99	2961.7			
Age 100+	2471.0			
Annual clinical vertebral fracture incidence rates per 100,000 persons (without intervention)				
Age 70–74	514	± 50%^b^	Triangular: ± 25%^b^	[3, 30]
Age 75–79	1106			
Age 80–84	2034			
Age 85–89	2331			
Age 90–95	3638			
Age 95–100	4369			
Age 100+	3645			
Relative risks of subsequent fractures associated with prior vertebral fractures				
Hip fracture	2.3	N/A	Gamma: 2.0–2.8^a^	[4]
Clinical vertebral fracture	4.4	N/A	Gamma: 3.6–5.4^a^	[4]
Relative hazards for mortality after a hip fracture				
Within a year	2.87	N/A	Gamma: 2.52–3.27^a^	[9, 15, 16]
Second year and beyond	1.73	N/A	Gamma: 1.56-1.90^a^	[9, 15, 16]
Proportions of excess mortality attributable to a fracture				
Hip fracture	0.25	N/A	Triangular: 0–0.5^b^	[9, 15, 16]
Clinical vertebral fracture	0	0.25 (Same as hip fracture)	N/A	[9]
Discount rates (%)				
Costs	2	N/A	Triangular: 0–4^e^	[18]
Quality-adjusted life-years	2	N/A		

^a^Based on 95% confidence intervals from meta-analyses

^b^Based on our own assumptions

^c^The upper bound of sensitivity analyses for adherence rates during the first year of treatment was set to be 0.8 and the lower bound was set symmetrically. For example, the adherence rate for teriparatide during the first year was 0.7 and the upper and lower bounds for sensitivity analyses were set to be 0.8 and 0.6, respectively. The ratio of upper bound to base case value (0.8/0.7) and lower bound to base case value (0.6/0.7) used in the first year were applied to the second year and beyond to obtain the upper and lower bounds for sensitivity analyses. The same principle was applied to alendronate. Estimated adherence or persistence rates exceeding 1.0 were considered to be 1.0

^d^Based on a previous cost-effectiveness analysis

^e^Based on the guideline

The reporting of this study followed the Consolidated Health Economic Evaluation Reporting Standards (CHEERS) statement and the recommendations for the conduct of economic evaluation in osteoporosis (Supplemental Table 2, 3) [31, 32].

### Model structure

We used TreeAge Pro Healthcare 2020 (TreeAge Software Inc., Williamstown, MA, USA) to program the model. Each cycle lasts 1 year, and every participant may sustain a hip or clinical vertebral fracture during each cycle. We only modeled the incidence of hip or clinical vertebral fractures because reliable epidemiological data regarding other osteoporotic fractures are lacking in Japan [15]. A participant can sustain only one fracture per cycle, and can have a maximum of two hip fractures and an unlimited number of clinical vertebral fractures over the entire time horizon. Details of the model structure may be found in our previous manuscripts [9, 15, 16].

### Efficacy of treatments

We compared the cost-effectiveness of sequential teriparatide/alendronate (i.e., daily subcutaneous teriparatide for 2 years followed by weekly oral alendronate for 8 years), compared with weekly oral alendronate monotherapy for 10 years. Data from a recent systematic review and network meta-analysis were used to obtain the efficacy of teriparatide and alendronate compared with placebo in reducing the risks of fractures among those at risk [20].

We took into account persistence and adherence with pharmacologic therapy. Persistence refers to “the duration of time from initiation to discontinuation of the therapy” and adherence refers to “the extent to which a patient acts in accordance with the prescribed interval and dose of a dosing regimen” [33]. Adherence rates were higher in clinical trials (mostly greater than 80%, as high as 100%) than observational studies that reflected actual clinical settings [34]. We estimated the relative effectiveness of treatments in the community by assuming a linear relationship between relative risk reduction and adherence [9, 15, 16].

We estimated the cumulative persistence rates with weekly bisphosphonates as approximately 55% and 10% at the end of first and seventh year, respectively, and the adherence rates with weekly bisphosphonates as 70.6% and 60.9% in the first and fifth year, respectively [35]. We assumed a linear decline in the adherence rates between the first year and fifth year. We assumed that those who took alendronate for 7 years continued to take alendronate for up to 10 years (i.e., no dropout from eighth year onward except for death) with the same adherence rate as the fifth year from the sixth year onward.

We estimated persistence rates of daily teriparatide at 68.0% and 51.6% at the end of the first and second years of use, respectively, based on an observational study in Japan [22]. This study did not provide the adherence rate, so we assumed the adherence rate to be 70.2% during the first year based on another study [21]. No data were available on the adherence rate beyond 1 year, so we assumed the adherence rate was 67.8% during the second year based on the estimated rate of decline in adherence to weekly alendronate as described above.

We assumed that alendronate was efficacious at reducing the risk of fractures from the first year through the tenth year, and that the risk of fractures after completing therapy returned to rates in the absence of alendronate over ten years in a gradual linear fashion (i.e., offset effect) [9, 15, 16]. Similarly, we assumed that teriparatide had efficacy from the first year through the second year and the risk for fractures returned to rates in the absence of teriparatide over 2 years, consistent with a recent systematic review [20]. In a deterministic sensitivity analysis, we assumed that the offset period of teriparatide was longer than that of alendronate. We assumed that teriparatide had efficacy from the first year through the end of the second year, and that the risk for fractures returned to rates in the absence of teriparatide after 3 years [9, 23]. We assumed the offset effect of teriparatide took effect if a participant took teriparatide but did not start alendronate afterward. For those who discontinued either alendronate or teriparatide before the pre-determined period (i.e., 2 years for teriparatide, 8 or 10 years for alendronate), the offset effects after discontinuation of therapy were assumed to be proportional to the length of the treatment periods. To keep the model parsimonious, we assumed that each individual obtained benefits of fracture prevention if she persisted in taking the treatment to the end of each cycle (i.e., 1 year).

### Transition probabilities

#### (a) Fracture rates

We modeled the annual incidence rates of hip fractures based on a recent study using Japan’s National Health Insurance Claims Database, which covers approximately 98% of all claims data in Japan [30]. The study did not report the rates of clinical vertebral fractures. We therefore used the incidence rates of hip and clinical vertebral fractures reported in one city and estimated the incidence rates of clinical vertebral fractures, assuming the same age- and sex-specific proportions of hip to clinical vertebral fractures applied to the values reported in the National Health Insurance Claims Database [3, 30]. As the target population was those who had prior vertebral fracture, we modeled increased relative risks of second and subsequent vertebral fractures and subsequent hip fracture associated with prior vertebral fracture [4].

#### (b) Mortality rates

Mortality rates were obtained from the abridged life table in 2018 [36]. The excess mortality rates after a hip fracture (either a first or a recurrent hip fracture) in the short term (within a year) and long term (starting in the second year and continuing lifelong) were included. We conservatively assumed that hip fracture events only contribute to 25% of the excess mortality, as comorbidities appear to play a large role [9, 15, 16]. We did not assume excess mortality associated with clinical vertebral fractures in the base case [9, 15, 37]. In an alternative scenario, however, we assumed the same excess mortality associated with clinical vertebral fractures as with hip fracture [16].

### Utilities

We used the EQ-5D based on noninstitutionalized population data in Japan to obtain age- and sex-specific baseline health state utility values [27]. We assumed that disutilities (i.e., loss in health-related quality of life) associated with hip and clinical vertebral fractures were highest in the year immediately following the fracture, but persisted lifelong [28, 29].

### Costs

We divided costs into formal healthcare sector and non-healthcare sector costs (Supplemental Table 1) [38]. We assumed that costs were identical regardless of age.

#### (a) Formal healthcare sector

We included the costs (the sums of payments by third-party payers and by patients out-of-pocket) of medications, prescription charges at pharmacy, physician visits, blood tests, DXA scans, and costs of medical treatments after fractures.

In Japan, drug prices covered by the public health insurance system are determined by the Ministry of Health, Labour and Welfare [39]. We used the cost of biosimilar teriparatide and assumed its efficacy to be the same as that of the brand version. In Japan, biosimilar teriparatide has been available since November 2019, with its price initially set to be 60% of the brand version. After April 2020, the price of brand product was decreased while that of biosimilar remained almost the same, making the price of biosimilar 70% of the brand version [7]. We estimated the cost of alendronate based on generic alendronate’s cost. We charged the cost of 3 months’ supply of teriparatide or alendronate (i.e., a single prescription filled) for those who discontinued teriparatide or alendronate within the first year. Costs of teriparatide or alendronate were proportional to adherence and persistence with the treatments.

Allowable charges based on the Japanese medical fee schedule for 2020 were used for the assumed costs of prescription charges at a pharmacy, physician visits (the cost incurred for the first visit was different from that for subsequent visits), blood tests and the fees for interpreting the results, and DXA scans [24]. Those who took teriparatide or alendronate had a physician visit every 3 months, as a prescription of medications beyond 3 months without a physician visit is not allowed in Japan. There did not appear to be a solid consensus regarding when and how frequently to perform blood tests, including renal function and calcium level, during treatment [5]. We assumed that those with teriparatide or with alendronate had a blood test twice a year. There also did not appear to be a consensus in terms of when patients should undergo a DXA scan after the initiation of osteoporosis treatments [5, 9, 12]. We charged the costs of a DXA scan at the end of the second, fifth, and tenth year.

We included the costs of medical resource use within 1 year after a fracture, including acute care and post-acute care, as future related medical costs. The cost of the treatments after hip and clinical vertebral fracture was based on a study using claims data in Japan [25]. The study provided the costs after a first clinical vertebral fracture and after a subsequent vertebral fracture. Our target population was those with prior clinical or morphometric vertebral fracture. To keep the model parsimonious, however, if a woman suffered from a first clinical vertebral fracture in the model, we applied the cost associated with the first clinical vertebral fracture regardless of whether the patient’s prior fracture history included a clinical or morphometric vertebral fracture. Future unrelated medical costs were not considered in this analysis, because we judged competing risks (for developing conditions other than a fracture) in an osteoporotic woman to be similar between sequential teriparatide/alendronate and alendronate monotherapy [9].

#### (b) Non-healthcare sectors

The annual long-term care costs for the “post-hip fracture” and “post-vertebral fracture” states were treated as non-healthcare sector costs, as public long-term care insurance is not a part of the universal healthcare insurance system in Japan. Long-term care costs in Japan include not only institutional care (e.g., long-term admission or short-term stay to a long-term care facility) but also community- and home-based care (e.g., adult day care, outpatient rehabilitation, home help, or home-visit nursing) [40]. A study using claims data in Japan estimated that the monthly cost of long-term care post-hip fracture averaged across those who started and did not start long-term care was ¥73,000 ($700) [26]. We therefore estimated the annual cost of long-term care post-hip fracture as ¥73,000*12=¥876,000 ($8340), which was charged across all participants in the “post-hip fracture” state until death. As there was a lack of data regarding long-term care costs associated with clinical vertebral fracture, we estimated those long-term care costs assuming the same proportion of annual long-term care to medical care costs for clinical vertebral fracture as applies to hip fracture. In the cycle in which a first hip or vertebral fracture occurred, half of the annual long-term care costs was charged.

### Model simulation and sensitivity analysis

For base case analyses, we ran the model with 100,000 iterations (100,000 individuals through the model one at a time). Next, we performed a special set of deterministic sensitivity analyses that varied the potential costs of future biosimilar teriparatide. Specifically, to determine the threshold costs that made sequential teriparatide/alendronate cost-effective under the pre-determined willingness-to-pay thresholds, we decreased the potential costs of biosimilar teriparatide in 5% increments compared to the current annual cost. We also simultaneously varied the annual incidence rates of hip and vertebral fracture from 50 to 150% of the base case, in 10% increments. We then performed deterministic (one-way) sensitivity analyses to evaluate the robustness of the results across a range of values for critical model parameters other than the cost of teriparatide (Table 1). We also examined two additional deterministic sensitivity analyses, in which (1) we assumed that the offset effect of teriparatide would be 3 years after a 2-year treatment period, and (2) we assumed the same excess mortality associated with clinical vertebral fractures as with hip fracture.

In addition, we performed probabilistic sensitivity analyses, in which parameter values were randomly selected from their probability distributions for uncertain key model inputs. Monte Carlo simulation was performed with 1000 simulations and 100,000 trials per simulation. In order to verify the model’s accuracy, we initially included a “no-intervention” arm to calculate mortality and fracture rates in the model.

## Results

### Base case analysis

#### Model validation

Our model predicted that without an intervention, the probabilities of dying by age 105 with different starting ages (i.e., 70, 75, or 80) were greater than 99%, consistent with those in the 2018 Japanese life table [36]. Our model also predicted that without an intervention, the lifetime probabilities of the study population having at least one hip fracture or one clinical vertebral fracture after the starting ages were approximately 35% or 69–72%, respectively.

#### Base case analysis

The ICERs of teriparatide/alendronate compared with alendronate were $282,300/QALY, $120,600/QALY, and $56,900/QALY at ages 70, 75, and 80, respectively. From a public healthcare sector perspective, the ICERs were similar to those from the combined public healthcare and long-term care sectors’ perspective and the conclusions remained the same (Table 2).

**Table 2:** Results of Base-Case Analyses at Various Ages of Therapy Initiation (ages 70, 75, and 80)

	Lifetime cost (US dollars, $1=¥105)	Quality-adjusted life-years (QALY)	Incremental cost-effectiveness ratio
From public healthcare and long-term care payer’s perspective (primary analysis)			
Age 70			
Alendronate monotherapy	$35,540	11.292	Comparator
Teriparatide/alendronate	$38,440	11.302	$282,300/QALY
Age 75			
Alendronate monotherapy	$35,340	8.846	Comparator
Teriparatide/alendronate	$37,890	8.867	$120,600/QALY
Age 80			
Alendronate monotherapy	$32,630	6.599	Comparator
Teriparatide/alendronate	$34,580	6.633	$56,900/QALY
From public healthcare payer’s perspective (sub analysis)			
Age 70			
Alendronate monotherapy	$11,900	See above	Comparator
Teriparatide/alendronate	$15,070	See above	$289,000/QALY
Age 75			
Alendronate monotherapy	$12,420	See above	Comparator
Teriparatide/alendronate	$15,490	See above	$138,700/QALY
Age 80			
Alendronate monotherapy	$12,240	See above	Comparator
Teriparatide/alendronate	$15,130	See above	$84,500/QALY

#### Deterministic sensitivity analysis

**Fig. 1:**
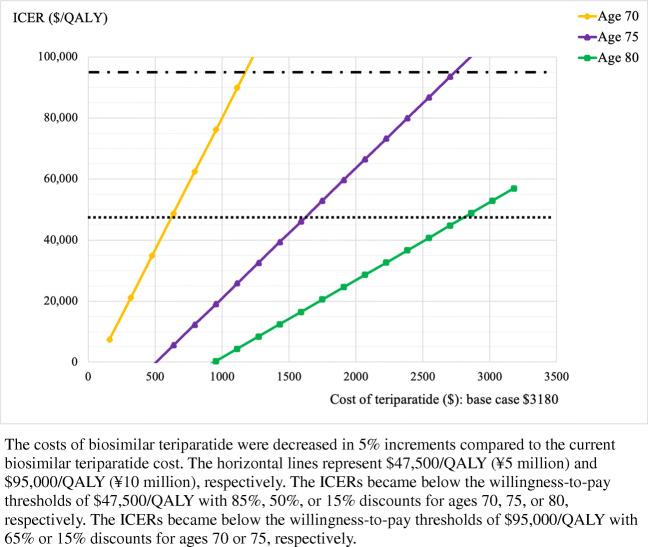
Results of deterministic sensitivity analyses varying the costs of teriparatide at ages 70, 75, and 80

When we decreased the potential annual costs of biosimilar teriparatide in 5% increments compared to the current biosimilar cost (i.e., ¥333,400 or $3180), sequential teriparatide/alendronate became cost-effective at a willingness-to-pay of ¥5 million ($47,500) per QALY with an 85%, 50%, and 15% discount at ages 70, 75, and 80, respectively (Fig. 1). When the annual incidence rates of hip and clinical vertebral fracture were ranged simultaneously from 50 to 150% in 10% increments compared with the incidence rates in the base case, the ICERs of sequential teriparatide/alendronate remained above the willingness-to-pay of ¥10 million ($95,000) per QALY at age 70. At age 75, the ICERs became less than the willingness-to-pay of ¥10 million ($95,000) per QALY with higher annual incidence rates of fracture (i.e., incidence rate ≥ 140% of base case). At age 80, the ICERs became less than the willingness-to-pay of ¥5 million ($47,500) per QALY with higher annual incidence rates of fracture (i.e., incidence rate ≥ 120% of base case). The ICER, however, exceeded the willingness-to-pay of ¥10 million ($95,000) per QALY with lower annual incidence rates of fracture (i.e., incidence rate = 50% of base case) (Supplemental Figs. 1 and 2).

Other than the cost of teriparatide or annual incidence rates of fracture, for cohorts with starting ages of 75 or 80, results were sensitive to changes in the efficacy of teriparatide or alendronate for fracture, the cumulative persistence rates of teriparatide or alendronate, or the adherence rate of teriparatide or alendronate. Results were insensitive to changes in parameter values for age 70. The ICERs of sequential teriparatide/alendronate became less than a willingness-to-pay of ¥10 million ($95,000) per QALY at age 75 with favorable parameter values. At age 80, the ICERs of sequential teriparatide/alendronate became less than a willingness-to-pay of ¥5 million ($47,500) per QALY with favorable parameter values, but also exceeded a willingness-to-pay of ¥10 million ($95,000) per QALY with unfavorable parameter values (Supplemental Figure 3).

In additional sensitivity analyses, in which we assumed that the offset effect of teriparatide lasted longer than the teriparatide treatment period, sequential teriparatide/alendronate became cost-effective at age 80. If we assumed the same excess mortality associated with clinical vertebral fractures as with hip fracture, the results were similar to the base case and the conclusions remained the same.

#### Probabilistic sensitivity analysis

**Fig. 2:**
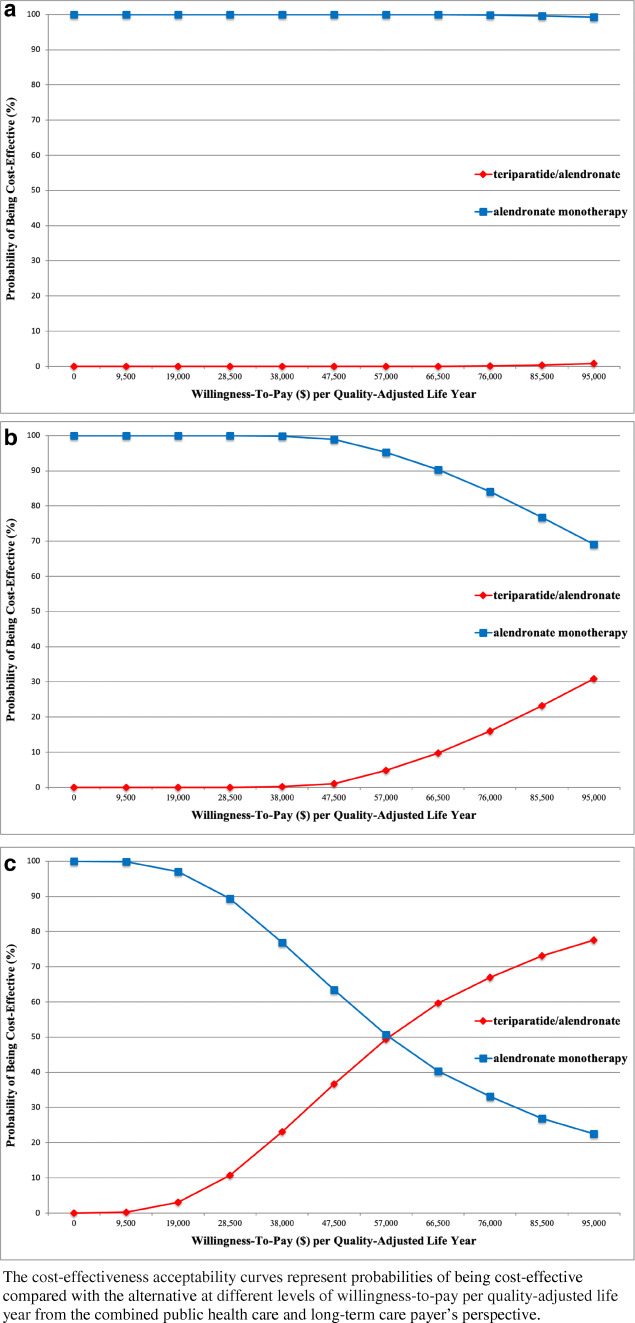
Results of probabilistic sensitivity analyses, **a**: age 70, **b**: age 75, **c**: age 80

The probabilities of teriparatide/alendronate being cost-effective compared with alendronate monotherapy were 0.0%, 1.1%, and 36.6% for ages 70, 75, and 80 respectively, at a willingness-to-pay of ¥5 million ($47,500) per QALY. At a willingness-to-pay of ¥10 million ($95,000) per QALY, the probabilities of teriparatide/alendronate being cost-effective were 0.8%, 30.9%, and 77.5%, for ages 70, 75, and 80, respectively (Fig. 2).

## Discussion

Although teriparatide is generally not considered to be a first-line treatment for osteoporosis, from the health economic standpoint teriparatide (including both daily and weekly versions) represented 20% of the total costs of medications used for osteoporosis in Japan in 2017 [5, 6]. We examined the cost-effectiveness of sequential daily teriparatide/alendronate compared with alendronate monotherapy among community-dwelling older osteoporotic women with prior vertebral fracture in Japan. In our model, without an intervention, the lifetime probabilities of a woman having a hip or vertebral fracture after the starting ages were approximately 35% or 69–72%, respectively, representing a high-risk population for osteoporotic fracture. Sequential teriparatide/alendronate was not cost-effective at any age examined, even with the recent availability of biosimilar teriparatide, at the conventionally accepted willingness-to-pay threshold (i.e., ¥5million, or $47,500/QALY).

In Japan, the Ministry of Health, Labour and Welfare determines and revises drug prices under the universal healthcare insurance system. The price of biosimilar teriparatide was initially set to be 40% discounted relative to the brand version when the biosimilar version became available on the Japanese market in November 2019. In April 2020, the prices of brand and biosimilar teriparatide were decreased by approximately 17% and 3%, respectively, making the price of the biosimilar 30% discounted relative to the brand product [7]. Sequential teriparatide/alendronate became cost-effective if the costs of biosimilar teriparatide were 85%, 50%, and 15% discounted relative to the current biosimilar price (or approximately 91%, 71%, and 51% discounted compared with the price of the brand version before November 2019) at ages 70, 75, and 80, respectively, at the willingness-to-pay of ¥5million ($47,500). At a willingness-to-pay of ¥10million ($95,000), sequential teriparatide/alendronate became cost-effective with discounts of 65% and 15%, respectively, (i.e., approximately 80% and 51% discounted compared with the price of the brand version before November 2019) at ages 70 and 75, and was cost-effective with no discount at age 80. Based on these results, it seems unlikely that teriparatide will be cost-effective at age 70 for the foreseeable future.

In an earlier analysis, we found that among community-dwelling older osteoporotic women with prior vertebral fracture in the USA, even with generic/biosimilar teriparatide availability, sequential teriparatide/alendronate would not be cost-effective unless the cost of generic or biosimilar teriparatide was heavily discounted with respect to the brand cost (i.e., 75%, 65%, and 70% discount at ages 70, 75, and 80, respectively) [9]. Parameter inputs differ markedly by setting (Supplemental Table 4), which shows the importance of conducting country-specific cost-effectiveness analyses.

In deterministic sensitivity analyses, we found that varying the efficacy of teriparatide or alendronate would impact the ICERs of sequential teriparatide/alendronate compared with alendronate monotherapy, which is not surprising. In deterministic sensitivity analyses, a relative risk of 0.16 was used for the lowest (i.e., most efficacious) value for efficacy of teriparatide for prevention of clinical vertebral fracture. Of note, this value is very similar to the relative risk of 0.17 reported in a secondary analysis of back pain findings from the global, multi-site Fracture Prevention Trial [41]. Persistence with teriparatide or alendronate also appears to influence ICERs substantially, consistent with the role of persistence noted in our previous work [15].

As evidence accumulates regarding the value of sequential therapy for the treatment of osteoporosis, cost-effectiveness analyses evaluating sequential therapy have also been performed [42]. In addition to our previous study comparing sequential teriparatide/alendronate with alendronate monotherapy [9], three more cost-effectiveness analyses regarding sequential therapy have been reported, all of which were performed in women in the US setting. Abaloparatide followed by alendronate was dominant (i.e., more effective and less expensive) compared with sequential teriparatide/alendronate and was cost-effective compared with alendronate monotherapy [23, 43, 44]. In our current study, however, abaloparatide was not included as it was not available in Japan at the time of this analysis.

We chose to evaluate cost-effectiveness from the combined public healthcare and long-term care payer’s perspective as a primary analysis, and from the public healthcare payer’s perspective as a sub-analysis, the latter being considered standard per Japanese guidelines [18]. The rationale behind this decision is that osteoporotic fracture leads to not only increased medical but increased long-term care expenditures, and we believed that in an older population, the economic burden on society caused by medical conditions/diseases is better evaluated by the sum of medical and long-term care expenditures, rather than medical expenditures alone [26, 45]. In this study, the ICERs of two perspectives turned out to be similar and the conclusions remained the same, even though long-term care represents approximately two-thirds of all costs.

We note several limitations. First, although the annual incidence rate of clinical vertebral fractures and the cost of long-term care after clinical vertebral fractures were key parameters, we estimated these values indirectly because of the lack of reliable data. Second, as the target population was those who had prior vertebral fracture, we modeled an increased risk of fracture associated with prior vertebral fracture. Bone mineral density (BMD) is also known to be a critical risk factor for fracture. However, we did not consider various BMD thresholds in our study, as the existing literature does not allow us to differentiate the increased risk associated with prior fracture from that associated with lower BMD (since the two are correlated). In a deterministic sensitivity analysis, we varied the annual incidence rates of hip and clinical vertebral fracture simultaneously from 50 to 150% of the base case to examine how these changes would affect the ICERs. By doing so, we have indirectly examined how changes in BMD thresholds would affect the ICERs*.* Third, we only included hip and clinical vertebral fractures and did not include other types of osteoporotic fractures, such as distal forearm or proximal humerus fractures. We believe, however, that including these other fracture types would have little influence on the overall results. Epidemiologic data from a Japanese city showed that hip and clinical vertebral fractures accounted for approximately 77% (ages 70–74), 84% (ages 75–79), 85% (ages 80–84), and 88% (ages 85 and older) of the four types of fractures (i.e., hip, clinical vertebral, distal forearm, and proximal humerus). In addition, among these four types of fractures, hip fractures are associated with the greatest medical and long-term care costs, reduced health-related quality of life in the first and subsequent years after the fracture, and excess mortality; while vertebral fractures are associated with medical and long-term care costs and reduced health-related quality of life in the first and subsequent years. In contrast, distal forearm and proximal humerus fractures are typically only associated with medical cost and reduced health-related quality of life in the first year after the fracture. Therefore, hip and clinical vertebral fractures are likely to be the key clinical events that need to be explicitly modeled. Fourth, to keep the model parsimonious, we did not include adverse events (e.g., hypercalcemia with teriparatide) [10, 34]. However, serious adverse events caused by teriparatide are considered to be rare and therefore were unlikely to impact the results of cost-effectiveness analyses [9]. Fifth, alendronate was prescribed after the completion of teriparatide in our analysis. However, another medication such as denosumab can be prescribed afterward instead of alendronate, which was beyond the scope of our analysis [9]. Finally, our results may be best applied to postmenopausal women in Japan, and may not generalize to women of other races/ethnicities or in other countries, or men.

Despite these limitations, our study has notable strengths. First, to our knowledge, this is the first economic evaluation to examine the cost-effectiveness of a teriparatide-based treatment strategy in Japan. Second, we incorporated the cost of biosimilar teriparatide that became recently (i.e., November 2019) available in Japan and then examined how further discounts of the costs of biosimilar teriparatide would affect cost-effectiveness. As in our previous study in the US setting, we identified that one of the main drivers of sequential teriparatide/alendronate not being cost-effective was the cost of teriparatide. Third, we incorporated medication persistence and adherence into the model and extensively examined how the changes in these parameters affect the ICERs in deterministic sensitivity analyses, as persistence and adherence rates have been known to be essential parameters in cost-effectiveness analyses regarding osteoporosis [9, 15].

In conclusion, among community-dwelling older osteoporotic women with prior vertebral fracture in Japan, sequential teriparatide/alendronate is not cost-effective compared with alendronate monotherapy at the ages examined, even with the availability of biosimilar teriparatide.

## Supplementary Information

ESM 1 (DOCX 37.0 kb)

ESM 2 (DOCX 480 kb)

## Data Availability

Non applicable

## Code availability

Non applicable
