# Supplementary material for: Cost-effectiveness of sequential daily teriparatide/weekly alendronate compared with alendronate monotherapy for older osteoporotic women with prior vertebral fracture in Japan
Source: Arch Osteoporos. 2021 Apr 17;16(1):72. doi: 10.1007/s11657-021-00891-z (PMC8053143; doi:10.1007/s11657-021-00891-z)
Supplement: Supplementary file 1 — (DOCX 37.0 kb) [file 11657_2021_891_MOESM1_ESM.docx]

**Supplemental Table 1: Impact Inventory**

| **Type of Impact** | **Perspective** | |
| --- | --- | --- |
|  | Public health care and  long-term care payer | Public health care payer |
| **Formal Health Care Sector** | | |
| **Health Outcomes (effects)** |  |  |
| Longevity | 🗹 | 🗹 |
| Health-related quality-of-life | 🗹 | 🗹 |
| Other (e.g., adverse events) | ☐ | ☐ |
| **Medical costs** | | |
| Medications | 🗹 | 🗹 |
| Physician visits | 🗹 | 🗹 |
| Blood tests | 🗹 | 🗹 |
| DXA scans | 🗹 | 🗹 |
| Future related costs (i.e., treatment for fractures) | 🗹 | 🗹 |
| Future unrelated medical costs | ☐ | ☐ |
| **Non−Health Care Sectors** | | |
| Cost of long-term care after fracture | 🗹 | ☐ |
| Cost of unpaid lost productivity due to fracture | ☐ | ☐ |

**Supplemental Table 2: The Consolidated Health Economic Evaluation Reporting Standards (CHEERS) statement**

**CHEERS checklist—Items to include when reporting economic evaluations of health interventions**

| **Section/item** | **Item no.** | **Recommendation** | **Reported on page no./ line no.** |
| --- | --- | --- | --- |
| **Title and abstract** |  |  |  |
| Title | 1 | Identify the study as an economic evaluation, or use more specific terms such as “cost-effectiveness analysis” and describe the interventions compared. | page 1, line 1- |
| Abstract | 2 | Provide a structured summary of objectives, perspective, setting, methods (including study design and inputs), results (including base-case and uncertainty analyses), and conclusions. | page 1, line 5- |
| **Introduction** |  |  |  |
| Background and objectives | 3 | Provide an explicit statement of the broader context for the study. Present the study question and its relevance for health policy or practice decisions. | page 2, left column, line 1- |
| **Methods** |  |  |  |
| Target population and subgroups | 4 | Describe characteristics of the base-case population and subgroups analyzed including why they were chosen. | page 2, right column, line 27- |
| Setting and location | 5 | State relevant aspects of the system(s) in which the decision(s) need(s) to be made. | page 2, right column, line 27- |
| Study perspective | 6 | Describe the perspective of the study and relate this to the costs being evaluated. | page 2, right column, line 38- |
| Comparators | 7 | Describe the interventions or strategies being compared and state why they were chosen. | page 3, left column, line 41- |
| Time horizon | 8 | State the time horizon(s) over which costs and consequences are being evaluated and say why appropriate. | page 2, right column, line 36- |
| Discount rate | 9 | Report the choice of discount rate(s) used for costs and outcomes and say why appropriate. | page 3, left column, line 11- |
| Choice of health outcomes | 10 | Describe what outcomes were used as the measure(s) of benefit in the evaluation and their relevance for the type of analysis performed. | page 2, right column, line 32- |
| Measurement of effectiveness | 11a | *Single study–based estimates*: Describe fully the design features of the single effectiveness study and why the single study was a sufficient source of clinical effectiveness data. | not applicable |
|  | 11b | *Synthesis-based estimates*: Describe fully the methods used for the identification of included studies and synthesis of clinical effectiveness data. | page 3, left column, line 13- |
| Measurement and valuation of preference-based outcomes | 12 | If applicable, describe the population and methods used to elicit preferences for outcomes. | not applicable |
| Estimating resources and costs | 13a | *Single study–based economic evaluation*: Describe approaches used to estimate resource use associated with the alternative interventions. Describe primary or secondary research methods for valuing each resource item in terms of its unit cost. Describe any adjustments made to approximate to opportunity costs. | not applicable |
|  | 13b | *Model-based economic evaluation***:** Describe approaches and data sources used to estimate resource use associated with model health states. Describe primary or secondary research methods for valuing each resource item in terms of its unit cost. Describe any adjustments made to approximate to opportunity costs. | page 3, left column, line 13- |
| Currency, price date, and conversion | 14 | Report the dates of the estimated resource quantities and unit costs. Describe methods for adjusting estimated unit costs to the year of reported costs if necessary. Describe methods for converting costs into a common currency base and the exchange rate. | page 2, right column, line 31- |
| Choice of model | 15 | Describe and give reasons for the specific type of decision-analytic model used. Providing a figure to show model structure is strongly recommended. | page 3, left column, line 28- |
| Assumptions | 16 | Describe all structural or other assumptions underpinning the decision-analytic model. | page 2, right column, line 25- |
| Analytic methods | 17 | Describe all analytic methods supporting the evaluation. This could include methods for dealing with skewed, missing, or censored data; extrapolation methods; methods for pooling data; approaches to validate or make adjustments (e.g., half-cycle corrections) to a model; and methods for handling population heterogeneity and uncertainty. | page 2, right column, line 25- |
| **Results** |  |  |  |
| Study parameters | 18 | Report the values, ranges, references, and if used, probability distributions for all parameters. Report reasons or sources for distributions used to represent uncertainty where appropriate. Providing a table to show the input values is strongly recommended. | page 4-5 (Table 1) |
| Incremental costs and outcomes | 19 | For each intervention, report mean values for the main categories of estimated costs and outcomes of interest, as well as mean differences between the comparator groups. If applicable, report incremental cost-effectiveness ratios. | page 7, right column, line 16-  page 8 (Table 2) |
| Characterizing uncertainty | 20a | *Single study–based economic evaluation*: Describe the effects of sampling uncertainty for estimated incremental cost, incremental effectiveness, and incremental cost-effectiveness, together with the impact of methodological assumptions (such as discount rate, study perspective). | not applicable |
|  | 20b | *Model-based economic evaluation*: Describe the effects on the results of uncertainty for all input parameters, and uncertainty related to the structure of the model and assumptions. | page 7, right column, line 23-  page 8, left column, line 22-  page 9 (Figure 1)  page 10 (Figure 2) |
| Characterizing heterogeneity | 21 | If applicable, report differences in costs, outcomes, or cost-effectiveness that can be explained by variations between subgroups of patients with different baseline characteristics or other observed variability in effects that are not reducible by more information. | not applicable |
| **Discussion** |  |  |  |
| Study findings, limitations, generalizability, and current knowledge | 22 | Summarize key study findings and describe how they support the conclusions reached. Discuss limitations and the generalizability of the findings and how the findings fit with current knowledge. | page 8, right column, line 7- |
| **Other** |  |  |  |
| Source of funding | 23 | Describe how the study was funded and the role of the funder in the identification, design, conduct, and reporting of the analysis. Describe other nonmonetary sources of support. | page 12, left column, line 7- |
| Conflicts of interest | 24 | Describe any potential for conflict of interest among study contributors in accordance with journal policy. In the absence of a journal policy, we recommend authors comply with International Committee of Medical Journal Editors’ recommendations. | page 12, left column, line 12- |

Husereau D, Drummond M, Petrou S, Carswell C, Moher D, Greenberg D, Augustovski F, Briggs AH, Mauskopf J, Loder E (2013) Consolidated Health Economic Evaluation Reporting Standards (CHEERS)–explanation and elaboration: a report of the ISPOR Health Economic Evaluation Publication Guidelines Good Reporting Practices Task Force. Value in health: the journal of the International Society for Pharmacoeconomics and Outcomes Research

**Supplemental Table 3: Osteoporosis-specific checklist: specific items to include when reporting economic evaluations on osteoporosis**

| **Item** | **Item no.** | **Recommendation** | **Reported on page no./ line no.** |
| --- | --- | --- | --- |
| Transition probabilities | 1 | Report the transition probabilities and how they were estimated (including increased fracture risk) | Page 6, left column, line 3-  Table 1 |
| Excess mortality after fractures | 2 | Describe approaches and data sources used for the excess mortality after fractures | Page 6, left column, line 21-  Table 1 |
| Fractures costs | 3 | Describe approaches and data sources used for fractures costs | Page 6, right column, line 38-  Table 1 |
| Fractures effects on utility | 4 | Describe approaches and data sources used for the effects of fractures on utility | Page 6, left column, line 35-  Table 1 |
| Treatment effect during treatment | 5 | Describe fully the methods used for the identification, selection, and synthesis of clinical effectiveness data (per fracture site) | Page3, left column, line 40-  Table 1 |
| Treatment effect after discontinuation | 6 | Describe fully the methods used for the treatment effect after discontinuation | Page 3, right column, line 32-  Table 1 |
| Medication adherence | 7 | Describe approaches and data sources used for modeling medication adherence | Page 3, right column, line 1-  Table 1 |
| Treatment costs | 8 | Describe approaches and data sources used for therapy costs | Page 6, right column, line 1-  Table 1 |
| Treatment side effects | 9 | Describe approaches and data sources used for costs and utilities effects of adverse events | Page 11, right column, line 6- |

Hiligsmann M, Reginster J-Y, Tosteson A, Bukata S, Saag K, Gold D, Halbout P, Jiwa F, Lewiecki EM, Pinto D (2019) Recommendations for the conduct of economic evaluations in osteoporosis: outcomes of an experts’ consensus meeting organized by the European Society for Clinical and Economic Aspects of Osteoporosis, Osteoarthritis and Musculoskeletal Diseases (ESCEO) and the US branch of the International Osteoporosis Foundation. Osteoporos Int 30(1):45–57

**Supplemental Table 4: Differences and similarities between the current analysis in the Japanese setting and the previous analysis in the U.S. setting***

|  | **Current analysis (Japan)** | **Previous analysis (U.S.)** |
| --- | --- | --- |
| **Modeling** | | |
| Comparison | teriparatide for 2 years followed by alendronate for 8 years vs. alendronate only for 10 years | teriparatide for 2 years followed by alendronate for 10 years vs. alendronate only for 10 years |
| Model | Markov microsimulation | |
| Target | hypothetical cohorts of community-dwelling osteoporotic women ages 70, 75, and 80 with prior vertebral fracture in Japan | hypothetical cohorts of community-dwelling osteoporotic white women ages 65, 70, 75, and 80 with prior vertebral fracture in the U.S. |
| Perspective | the combined health care and long-term care sectors | societal |
| Time horizon | Lifetime | |
| The willingness-to-pay thresholds in the base case | $47,500 per QALY (2020 U.S. dollars) | $150,000 per QALY (2018 U.S. dollars) |
| Discount for costs and health benefits | 2% | 3% |
| Base case iterations | 100,000 | |
| **Fracture** | | |
| Osteoporotic fractures included | Hip and clinical vertebral fractures | Hip, clinical vertebral, wrist, and other fractures |
| Annual incidence per 100,000 persons, hip fracture, age 70-74, 75-79, 80-84 (general population without intervention) | 158, 362, 831 | 394, 793, 1447 |
| Annual incidence per 100,000 persons, clinical vertebral fracture, age 70-74, 75-79, 80-84 (general population without intervention) | 514, 1106, 2034 | 473, 523, 622 |
| Probabilities of having at least one hip or vertebral fracture after the starting age of 70 in the model | 35% (hip), 71% (vertebral) | 59% (hip), 56% (vertebral) |
| **Cost** (2020 U.S. dollars for the Japanese study, 2018 U.S. dollars for the U.S. study) | | |
| The proportions of brand and generic/biosimilar versions of medications included in analyses | Biosimilar only for teriparatide; generic only for alendronate | Combined brand (10%) and generic/biosimilar (90%) for teriparatide; combined brand (10%) and generic (90%) for alendronate |
| Cost of brand teriparatide | Not applicable | $27,618, approximately 5.4 times as high as the price of the identical counterpart in Japan as of 2018 |
| Cost of biosimilar (or biosimilar/generic) teriparatide | $3180 | $19,332 |
| Cost of brand alendronate | Not applicable | $1276 |
| Cost of generic alendronate | $83 | $86 |
| Treatment cost for hip fracture | $16,440 | $29,986 |
| Treatment cost for clinical vertebral fracture | $4000 (first), $8020 (subsequent) | $8325 |
| Annual long-term care cost for “post-hip fracture” state | $8340 | $2577** |
| Annual long-term care cost for “post-clinical vertebral fracture” state | $2030 | Not applicable |
| What long-term care covers | not only institutional care but also community- and home-based care (e.g., adult day care, outpatient rehabilitation, home help, or home-visit nursing) | institutional care |
| **Treatment** | | |
| Relative risk of hip fracture and clinical vertebral fracture with teriparatide | 0.35 and 0.23, respectively | 0.42 and 0.30, respectively |
| Relative risk of hip fracture and clinical vertebral fracture with alendronate | 0.64 and 0.50, respectively | 0.45 and 0.50, respectively |
| Persistence and adherence with teriparatide (12 months) | 68% and 70%, respectively | 63% and 54%, respectively |
| Persistence and adherence with alendronate (12 months) | 55% and 71%, respectively | 39% and 31%, respectively |
| **Utility** | | |
| Baseline utility | 0.810 (age 70-74), 0.771 (age 75-79), 0.769 (age 80-84) | 0.771 (age 70-79), 0.724 (age 80-89) |
| Disutility (multiplier) | 0.776, 0.855 for hip fracture (first year, beyond first year), 0.724, 0.868 for clinical vertebral fracture (first year, beyond first year) | |

***** Mori T, Crandall CJ, Ganz DA (2019) Cost‐Effectiveness of Sequential Teriparatide/Alendronate Versus Alendronate‐Alone Strategies in High‐Risk Osteoporotic Women in the US: Analyzing the Impact of Generic/Biosimilar Teriparatide. JBMR Plus 3 (11)

** We conservatively assumed that hip fractures themselves were directly responsible for only 25% of long‐term‐care placements in the U.S. setting.
