# Supplementary material for: Cost-effectiveness of sequential daily teriparatide/weekly alendronate compared with alendronate monotherapy for older osteoporotic women with prior vertebral fracture in Japan
Source: Arch Osteoporos. 2021 Apr 17;16(1):72. doi: 10.1007/s11657-021-00891-z (PMC8053143; doi:10.1007/s11657-021-00891-z)
Supplement: Supplementary file 2 — (DOCX 480 kb) [file 11657_2021_891_MOESM2_ESM.docx]

**Supplemental Figure 1: Results of Deterministic Sensitivity Analyses varying the Annual Incidence Rates of Fractures at ages 75 or 80**

**
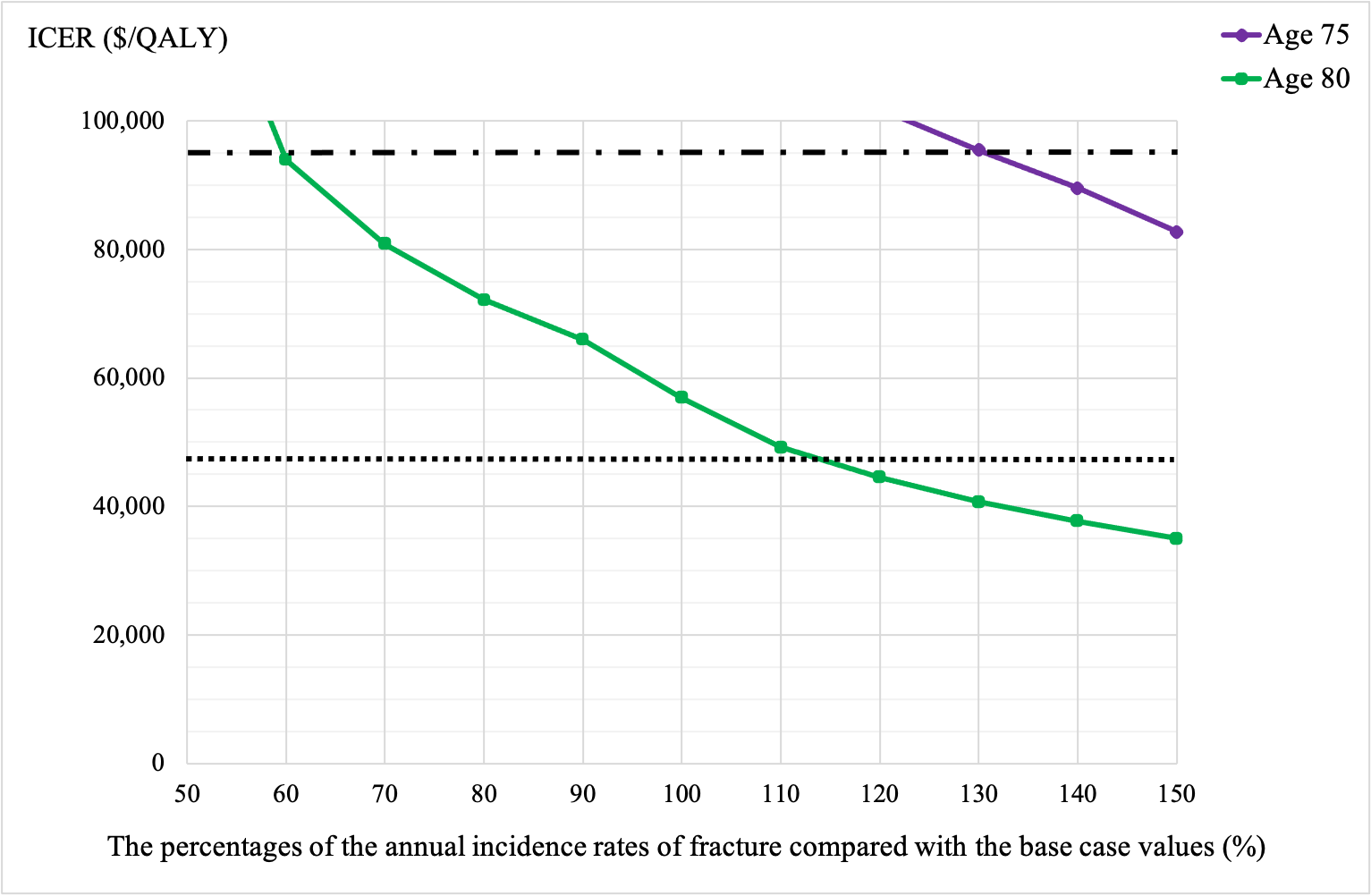
**

The figure presents the incremental cost-effectiveness ratios (ICERs) of sequential teriparatide/alendronate compared with alendronate monotherapy as hip and clinical vertebral fracture incidence rates are simultaneously ranged from 50% to 150% of their base case values in 10% increments. The horizontal hashed lines represent the pre-specified willingness-to-pay thresholds of $47,500 (¥5 million) and $95,000 (¥10 million) per quality-adjusted life year, respectively. The ICERs did not become below the pre-specified thresholds of willingness-to-pay at age 70.

**Supplemental Figure 2: The Lifetime Probabilities of Fracture varying the Annual Incidence Rates of Fractures at ages 70, 75, or 80**


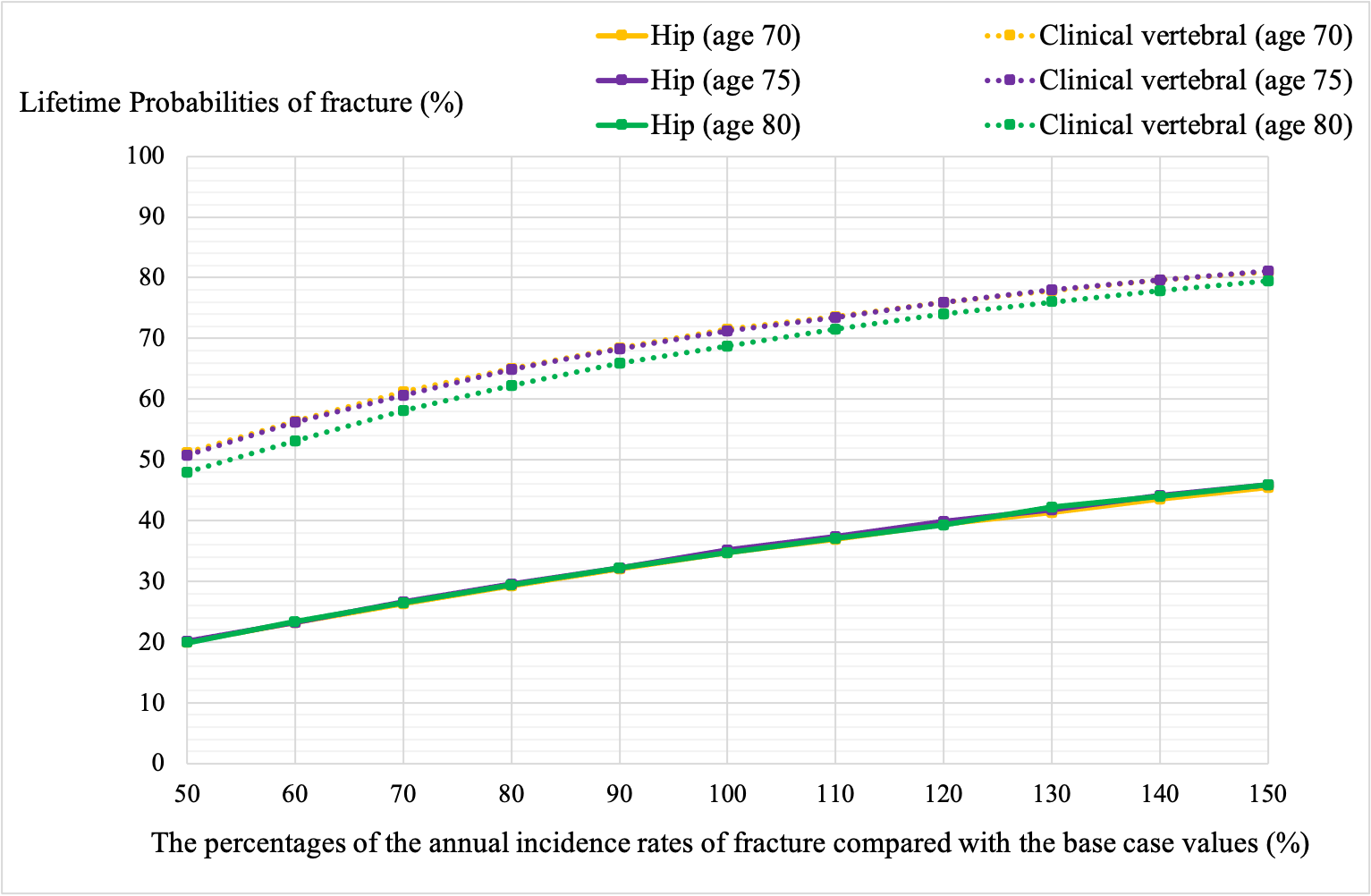


The figure presents lifetime probabilities of hip or clinical vertebral fractures, when the annual incidence rates of hip and vertebral fracture were simultaneously ranged from 50% to 150% of their base case values in 10% increments. The trajectories are superimposed for hip fracture at ages 70, 75, and 80, and for clinical vertebral fracture at ages 70 and 75.

**Supplemental Figure 3: Results of Deterministic Sensitivity Analyses Other Than Costs or Annual Incidence Rates of Fractures, a) age 75, b) age 80**

**
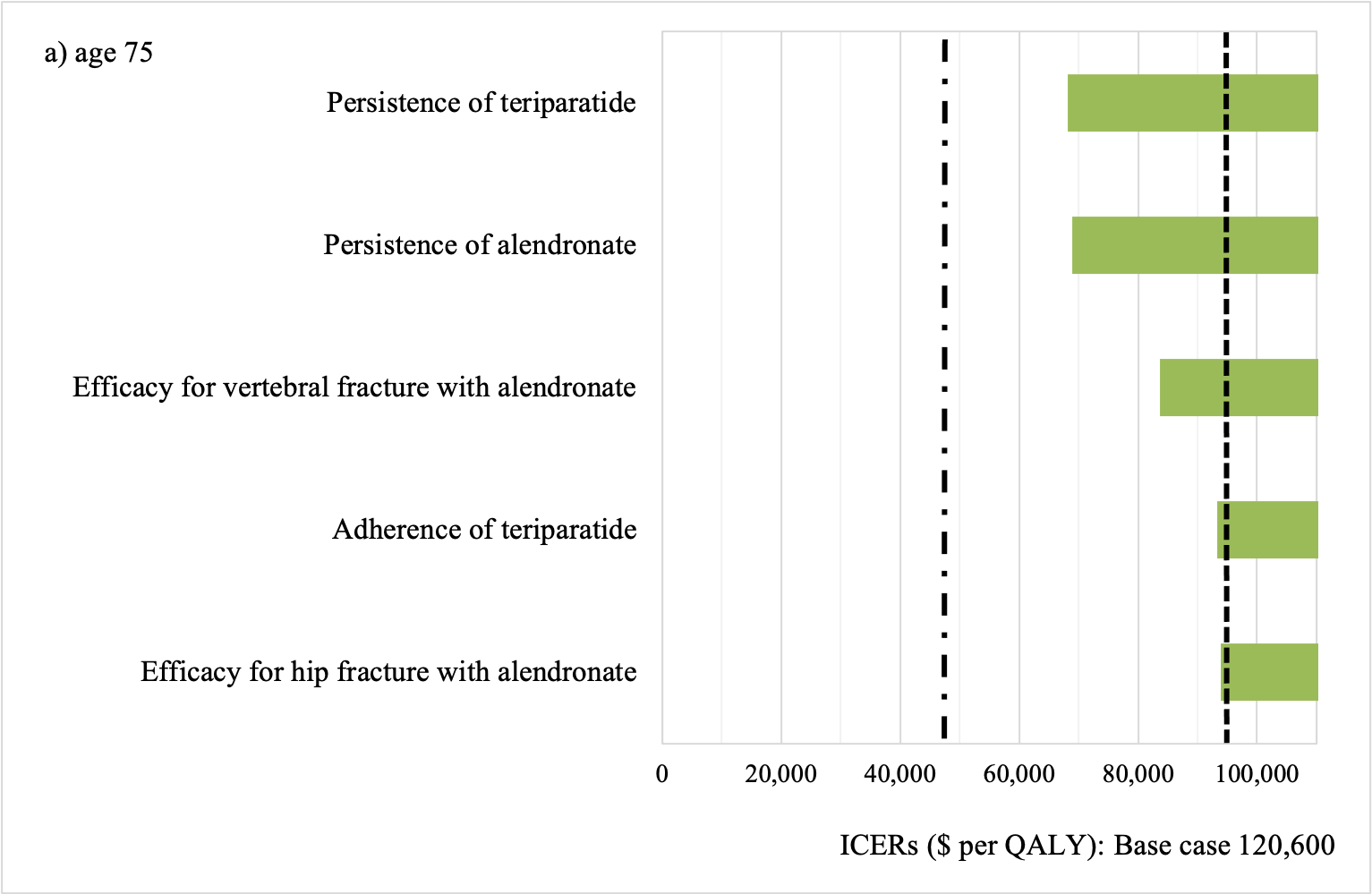
**

**
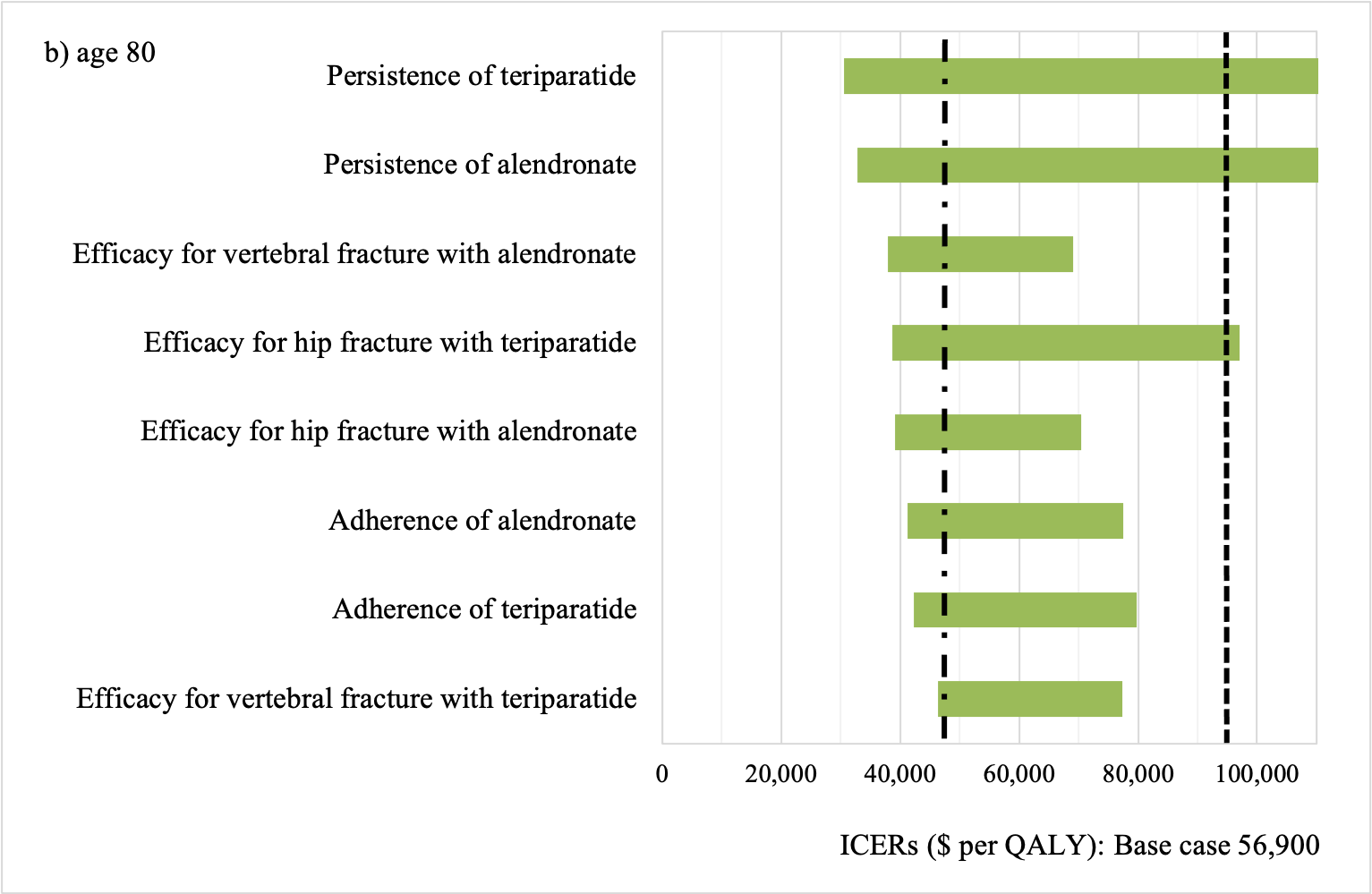
**

The figures present the incremental cost-effectiveness ratios (ICERs) of sequential teriparatide/alendronate compared with alendronate monotherapy, when the indicated model parameters are varied across their ranges from the combined public health care and long-term care payer’s perspective. The vertical hashed lines represent the pre-specified threshold of willingness-to-pay of $47,500 (¥5 million), and also $95,000 (¥10 million) per quality-adjusted life year. The ranges of each parameter are presented in Table 2. We present the results of three largest changes in ICERs. The results at age 70 are not presented as no changes in parameters make the ICER less than the willingness-to-pay thresholds.
